# Long COVID Patients’ Perceptions of Social Support in Their Work and Personal Lives: A Qualitative Study

**DOI:** 10.3390/healthcare13131568

**Published:** 2025-06-30

**Authors:** Willi L. Tarver, Xiaodan Hu, Sarah R. MacEwan, Alice A. Gaughan, Ann Scheck McAlearney

**Affiliations:** 1 Department of Internal Medicine, Division of Cancer Prevention and Control, The Ohio State University Wexner Medical Center, Columbus, OH 43202, USA; willi.tarver@osumc.edu; 2 Center for the Advancement of Team Science, Analytics, and Systems Thinking in Health Services and Implementation Science Research (CATALYST), College of Medicine, The Ohio State University, Columbus, OH 43202, USA; xiaodan.hu@osumc.edu (X.H.); sarah.macewan@osumc.edu (S.R.M.);; 3 Department of Internal Medicine, Division of General Internal Medicine, The Ohio State University Wexner Medical Center, Columbus, OH 43210, USA; 4 Department of Family and Community Medicine, The Ohio State University Wexner Medical Center, Columbus, OH 43210, USA

**Keywords:** Long COVID, social support, patient perspectives, qualitative methods

## Abstract

**Background:** The onset and persistence of Long COVID can lead to cognitive and functional impairment, contributing to illness-induced employment and work disparities. Understanding how social support influences these issues can inform care strategies and support continued workforce participation. **Objectives**: This study explored perceptions of social support among patients with Long COVID. **Methods**: Semi-structured interviews were conducted with 21 patients receiving care at a post-COVID recovery clinic. Patient perspectives on social support in their work and personal lives were analyzed using both inductive and deductive thematic analysis. Findings were organized under the following five dimensions of social support theory: tangible support, emotional support, informational support, appraisal support, and belonging support. **Results**: Patients received positive tangible, emotional, and informational support from family, friends, and credible sources. However, patients also described receiving negative appraisal support from their personal lives and workplaces when others misunderstood the scope and duration of their limitations due to Long COVID. This negative appraisal support often labeled them as lazy or underperforming, leading to both personal and professional challenges to their self-esteem. Regarding companionship support, participants reported challenges keeping in touch with others and being less social. **Conclusions**: Social support impacts Long COVID patients’ abilities to cope with the trauma of their experiences. Understanding the sources of and barriers to social support for Long COVID patients may inform strategies to enhance their care and well-being. Future interventions should offer opportunities for family, friends, and employers of Long COVID patients to learn about what it means to live with the illness.

## 1. Introduction

Since the first case of SARS-CoV-2 infection (COVID-19) was identified in December of 2019, it developed into a major pandemic causing significant morbidity and mortality. As of June 2025, more than 777 million cumulative cases of infection have been identified globally, leading to more than 7 million deaths [1]. While the majority of patients diagnosed with COVID-19 experience short-term mild-to-moderate illness, which may require careful monitoring, an estimated 31–69% of individuals will experience prolonged symptoms after the initial recovery period for the disease [2,3]. This condition involving long-term effects is known as Long COVID and is also referred to as post-acute COVID-19 syndrome, long-haul COVID-19, post-COVID conditions [PCC], and chronic COVID [4,5]. Patients themselves coined the term “Long COVID” to represent their experiences and bring attention to the persistence and heterogeneity of symptoms. This term challenges common assumptions about COVID-19 prevalent in the early pandemic that often persisted despite patients’ lived experiences and testimonies, subsequently influencing how the condition has been conceptualized [4,6].

Uncertainty about the definition of Long COVID persists as various pathophysiological mechanisms are putative. No single, definitive accessible biomarker currently exists for diagnosis, and changes have emerged in the natural history of this condition due to reasons including the evolution of viruses, novel therapeutics, and vaccination [7,8]. Considering these factors, we adopt the broad definition of Long COVID provided by the National Academies of Sciences [9], describing it as “…an infection-associated chronic condition (IACC) that occurs after SARS-CoV-2 infection and is present for at least 3 months as a continuous, relapsing and remitting, or progressive disease state that affects one or more organ systems.”

Common symptoms for Long COVID include fatigue, dyspnea or difficulty breathing, arthralgia or joint pain, cognitive impairment, anxiety, and depression [10,11]. Due to a constellation of physiological and psychological symptom combinations, one striking characteristic of Long COVID is functional impairment, which reduces individuals’ abilities to perform activities they could previously accomplish in their daily lives, including working [7]. These impairments, particularly those impacting cognition and physical stamina, can hinder individuals’ capacities to meet workplace demands. Notably, Long COVID has been associated with employment disparities as a result of cognitive symptoms that interfere with daily functioning [12,13]. Individuals living with this condition have been found to be disparately impacted by illness-induced employment and work disparities, as they experience higher rates of unemployment and reduced likelihood of full-time employment [12,13,14,15]. For instance, a US nationwide survey found that 12.3% of individuals with Long COVID reported being unemployed, compared to 8.7% of those without the condition. Additionally, only 45.5% of those with Long COVID worked full-time, compared to 55.2% of those without the condition [13]. Furthermore, qualitative research into patients’ experiences revealed in depth that functional impairment and changes in employment have led to work, income, and insurance-related stress that impact emotional well-being, resulting in feelings of loss regarding one’s self-identity, and fear of judgment in the workplace [16].

Given the compounded negative impacts of Long COVID on work and daily routine, patients also reported increased levels of loneliness [17]. In the early phases of the pandemic, this may have partly been due to public health efforts to reduce the spread of COVID-19 which emphasized self-isolation, social distancing, and self-quarantine. These measures may have had mental health implications that have not yet been sufficiently acknowledged. For example, research has found higher rates of depression, anxiety, and loneliness among individuals experiencing self-isolation compared to those who do not [18]. These issues could be particularly pronounced among Long COVID patients who may have been disproportionately subjected to longer periods of isolation. Even after formal quarantine measures were relaxed due to the pandemic, social exclusion and isolation continued to limit the social support received by those with Long COVID, critically impacting their overall well-being [17]. While social support from friends and family members has been shown to help individuals with Long COVID to return to daily activities and maintain psychological resilience [17], less is known about how social support, or the lack thereof, impacts employment outcomes and well-being for this population.

Current Long COVID research has primarily focused on the medical aspects of this illness, such as improving our understanding of the pathophysiology of this condition and identifying effective strategies for diagnosis and treatment [18,19]. However, less consideration has been paid to factors that may buffer or mitigate the negative impacts of Long COVID and improve the well-being and quality of life among those experiencing illness-induced disparities across social, employment, and health domains. Social support has been found to consistently benefit well-being and quality of life by providing people with means to avoid or mitigate their exposure to stressors [20]. For example, social support may influence cognitive processes and lead to individuals being less likely to perceive a situation as threatening [21]. In addition, social support can lead to proactive coping where stressors can be anticipated in advance and lead to efforts to prevent or minimize them [22]. In a context specific to Long COVID patients, social support has been shown to act as a buffer to worrying about COVID-19, as well as about psychological health [23]. Social support-related factors have also led to improvements in sleep, depressive symptoms, anxiety symptoms, and irritability in COVID-19-positive patients [24,25].

While social support has been found to be protective against some of the negative effects on mental health that patients with Long COVID may experience, we know little about the specific sources of social support for these patients and the impacts of this support on their experiences in personal and workplace-specific settings. Patients attempting to manage Long COVID symptoms need support beyond medical care which may involve social support from their immediate social circles. Therefore, our study explored Long COVID patients’ perspectives about the types, sources, and perceived helpfulness of the social support they reportedly received. Understanding patients’ perspectives about social support can have important implications for improving their quality of life, informing the development of strategies to support ongoing care, and identifying factors that may reduce the employment challenges they face.

## 2. Social Support Framework

This study takes into consideration the way social support influences the experiences of patients with Long COVID in both their work and personal lives. We focus primarily on functional social support, which represents the particular functions that are provided or perceived to be available through social relationships [26,27,28,29]. The systems approach views social relationships as a network embedding each individual in the family and close relationships, the community, and the society [27]. Consistent with this definition, social support can come from various sources, including family, friends, coworkers, and supervisors [30].

There are five overarching dimensions through which individuals can provide social support: (1) tangible support, (2) emotional support, (3) informational support, (4) appraisal support, and (5) belonging support [31,32,33]. *Tangible support* (or instrumental support) involves material aid, service, or practical help such as performing assigned work for others or assisting with physically demanding tasks [31,34]. *Emotional support* encompasses verbal or non-verbal communication to express empathy, trust, caring, hope, and love [28,31]. *Informational support* is defined as any advice, suggestions or facts provided during a time of stress that can help a person problem-solve and overcome a challenge [31,34]. *Appraisal support*, or esteem support, includes providing information, affirmation, and feedback that would help one cope with threats to self-esteem or self-evaluation [32,33]. Lastly, *belonging support* (companionship support) represents a sense of belonging and includes having others to engage with in social activities [31]. This five-dimension framework is an extension of the original theory of social support, which did not include belonging support [28,29]. Our study seeks to explore and describe Long COVID patients’ perspectives about their experiences, focused on their receipt of these forms of support. Accordingly, we address the following research questions:

(1) What types of social support do Long COVID patients receive and have access to that fit within the five social support dimensions (i.e., tangible support, emotional support, informational support, appraisal support, and belonging support)?
(2) What forms of social support are helpful or not helpful for Long COVID patients?
(3) What are the unmet social support needs of Long COVID patients in work and personal contexts?

## 3. Methods

### 3.1. Study Design

We conducted semi-structured interviews with patients receiving care at a post-COVID recovery clinic within an academic medical center located in the state of Ohio. Participants were informed of the study’s purpose, their rights, and the research team’s intent to audio record the interview. With the participants’ consent, all interviews were audio recorded and transcribed verbatim. Two male and three female research team members conducted the interviews. They were PhD- or MS-trained health services researchers affiliated with CATALYST at the time of the study. Ethical approval was obtained from the Institutional Review Board (IRB) of The Ohio State University (IRB protocol number: 2020B0288).

### 3.2. Recruitment

Patients with Long COVID who self-reported doing well before having COVID-19 and were still significantly impacted three or more months post-acute infection were eligible for study participation. In addition, eligible patients had to speak English, be 18 years of age or older, and sought care at the post-COVID recovery clinic. Interviews were conducted by phone or videoconference in August and September of 2022. Interested patients were contacted by the research team via email, where they were provided with further details about the study, questions were answered, their interest was confirmed, and the interview was scheduled.

### 3.3. Data Collection

The interview guide (see Supplementary Materials) facilitated discussion around main topics including, but not limited to, the following: (1) perspectives on obtaining a diagnosis of Long COVID; (2) perspectives on support from family, friends, and healthcare providers; and (3) perspectives on stigma from having Long COVID. Participants received a $25 electronic gift card after their interview in appreciation for their time.

### 3.4. Analytical Methods

Transcripts were coded and analyzed by a total of five coders using ATLAS.ti (Version 25.0.1), a qualitative data analysis package. All coding was conducted manually without the use of auto-coding or frequency-analysis functions. We explored patients’ perspectives about social support using both inductive and deductive thematic analysis [35]. We first created a preliminary coding dictionary based on topics from the semi-structured interview guide. A single transcript was then chosen for research team members to use to independently code using the preliminary coding dictionary. This coding team met to perform the following: (1) compare the application of codes; (2) discuss their consistent use among coders; (3) refine codes and definitions; and (4) add relevant new codes for themes that emerged during coding and resulted in a refined coding dictionary. This process was repeated for a second transcript using the refined coding dictionary. The remaining transcripts were then divided among team members to code using the revised coding dictionary. Team members continued to meet frequently to address questions about coding and resolve disagreements about application of codes through team consensus. Specifically, we conducted weekly one-hour meetings during preliminary coding to discuss code definitions, application of codes, and emergent codes. Changes to the coding dictionary and resolution of conflicting code assignments were made by reaching consensus among all coders. These frequent meetings ensured consistency of coding across transcripts.

Within the revised coding dictionary (See Supplementary Materials), several codes focused on the following patient perspectives about social support: (1) support from family and friends; (2) support from providers; (3) impact on mental health; attending support groups; (4) impact on employment or school; (5) isolation; and (6) information sources. In a round of secondary coding, reports with these coded quotations across all transcripts were reviewed by the research team to characterize themes and subthemes that represented Long COVID patients’ perspectives about social support.

## 4. Results

We interviewed 21 patients for the study (Table 1) who had a mean age of 47.6 years (range: 19–68). Approximately three-quarters of our sample was female. Interviews lasted an average of 49.5 min (range: 16.97–84.48).

Participants shared their experiences with Long COVID, including how the condition impacted their work and personal lives. While some reported having received tangible, emotional, and informational social support from family, friends, and the workplace, negative appraisal support and barriers to seeking belonging support were also described. Our results highlight the unique challenges Long COVID patients face, which can undermine their self-esteem as well as how they identify both personally and professionally. Across interviews, we characterized five themes corresponding to the five dimensions of the Social Support Framework [31,32,33] as well as sub-themes within each theme. A summary of these themes, subthemes, their descriptions, and representative quotations supporting these characterizations is presented in Table 2.

### 4.1. Tangible Support

Participants self-reported receiving helpful tangible support from four sources, which emerged as distinct subthemes in our analysis: (1) family, (2) friends, (3) the workplace, and (4) neighbors. Several participants shared challenges with physically demanding daily activities. Family was described as the source that provided regular aid to frequently help participants with tasks such as lifting things, managing household responsibilities, and driving. Tangible support from a spouse was reported as particularly important, as one participant explained, “*My husband, it’s been very practical. I mean, he cooks, he cleans, he does laundry, he takes care of, you know, the yard, he goes to work every day. I mean, literally, it’s, if I mean, he has taken over 80% of the chores that I used to do*” (P07). Participants also received similar tangible support from friends. Such support was reportedly less frequent and more oriented toward smaller tasks when compared to the tangible support received from family members. As one participant shared, *“There were times where they’ve [my friends] come over and like helped me do tasks that are just a little physically overwhelming for me. Like putting stuff up in the attic, or anything that requires like more stamina that I just don’t have*” (P18).

Some participants described tangible support provided by their employer, including allowing flexibility in work location and hours, which enabled them to manage their Long COVID symptoms and well-being while maintaining productivity. One participant reflected, “*When they [my company] went back to the office, I was allowed with the doctor’s note to say, I have to work at home. They also were able to allow me to block off my calendar towards the end of the day where my brain fog was the worst*” (P03). The tangible support received extended beyond our participants’ immediate social circles. Some shared instances where they received spontaneous assistance with caring for their family or property from their neighbors. This also included receiving neighbors’ assistance with farm work on which their livelihoods depended. For example, one participant described, “*The neighbor across the road, she came over with her daughters to help my mom set up all the pens to get ready for lambing. Her husband came over and helped get the issue–I had some issues on a skid steer that went bad. He fixed all that*” (P13).

While participants acknowledged how important it was to receive tangible support, they also noted concern about being a burden. One participant shared that while her husband had been very supportive, it was not easy for him: *“… it’s also a lot of added pressure and a lot of added burden. A lot, a lot of extra on his plate that didn’t used to be there. And he’s not young, he’s 65. You know, he’s not a young man, so it’s not easy for him”* (P07).

### 4.2. Emotional Support

The Long COVID patients we interviewed reported receiving positive emotional support from the following: (1) friends, (2) family, and (3) the workplace, which constituted the three subthemes of our analysis. Participants reported that their friends had provided considerable emotional support, such as when friends checked in on their well-being or were flexible and understanding about changes in social activity arrangements. As one participant noted, “*They’re understanding. Like if I have to last minute cancel something, I ran out of energy or I have a headache that I’ve been dealing with all day*” (P02).

Emotional support from family was manifested through verbal encouragement as well as understanding about difficulties with emotional control due to Long COVID symptoms. For example, one participant commented, “*She [my wife] has urged me along the way and encouraged me and has been very understanding. Like when I’ve lost my temper with her, that kind of thing, because I couldn’t keep up with a thought process. And, you know, so incredibly encouraging*” (P16).

In several instances, participants described emotional support they received from the workplace. Some employers were highlighted for fostering a trusting and safe environment, while also showing understanding about the fatigue and cognitive impairments experienced by employees living with Long COVID symptoms. One participant explained, “*He [my boss] has not been intrusive, but he has created an environment where I feel safe enough that I can let him know, hey, this is what’s going on with me, here are the struggles that I’m currently having*” (P01).

When emotional support was lacking or perceived to be negative, Long COVID patients attributed this to family, friends, and/or coworkers having unrealistic expectations of them and lacking insight about the experience of having Long COVID. In some cases, participants reported that social support waned over time. One participant shared, *“No one thought 15 months out I would still be the same. So, like I said, I had tons and tons of support in the beginning for a good six months. That is long. I was receiving cards. People would come over and put messages on the doors. It was just very supportive. And now it’s just I feel kind of forgotten”* (P11). Similarly, emotional support from the workforce was reported to decrease overtime for some participants. As one interviewee reflected, “*When I was working with HR [human resources] regarding my leave, they were very much like, oh, how are you feeling? But ever since I’ve returned to work, I’ve needed to change things around. Like when I first was returning, I told you I had all those, like I was seeing all the specialists. And I would have easily five to six appointments a week. But when I was returning, I was like, hey, for the next couple weeks since these are pre-existing appointments, can I change around my schedule? Is it ok if I take extended lunches? And they were like, absolutely not. Business can’t support that*” (P02).

### 4.3. Informational Support

Our study participants reported receiving informational support from: (1) credible sources, and (2) support groups, which served as the two subthemes. Trusted news websites and medical/peer-reviewed journals were reported to have provided up-to-date medical information about COVID-19 and medications. As one participant commented, “*I trust the New York Times, but just recognizing that there’s a lot of misinformation out there too. So, I think just making sure or giving patients the opportunity to get plugged into reputable, good sources of information about COVID and post COVID*” (P01).

In addition to medical and health-related information from specialized sources, participants also expressed the need to learn about other patients’ lived experiences with Long COVID through support groups. While less formal, this type of informational support reportedly provided real-time insights, a greater variety of perspectives, and highly personalized information that could help study participants navigate Long COVID. One participant explained, “*I belong to like three of them [support groups]. And there, you know, just to bounce ideas, you know, to hear about what’s helping some people, what’s not helping some people, different things that they’re trying. And, you know, post links to different studies that are being done. But just, you know, kind of a good way to just keep up with things*” (P07). Study participants also noted the potential downsides of support groups such as the emotional toll of hearing others repeatedly discuss their symptoms. As one participant reflected, “*I do have to be careful because, honestly, they can be really depressing, you know, because everyone there is in a bad way*” (P07).

### 4.4. Appraisal Support

Two subthemes emerged within the appraisal support dimension, both characterized by negatively framed evaluations related to one’s performance in the following: (1) personal life and (2) the workplace. Unhelpful negative appraisals include instances of patients being explicitly or implicitly labeled as overreacting, lazy, or unreasonable due to others’ lack of understanding about the symptoms and chronic nature of Long COVID. These appraisals suggest that individuals with Long COVID face challenges to their self-esteem both personally and professionally. Several participants commented on the hurt, frustration, and helplessness they felt when being negatively appraised by those from their close social circles. One participant explained, “*I have a lot of friends who, you know, tell me that too, tell me just, just get out of bed and go for walk. Damn, just do something! And you know, you can hear that tone in their voice. They’re just kind of contempt*” (P07). Another reflected, “*They forget I’m in pain and I have these other symptoms. They see me on the oxygen, but the rest of it’s invisible. And there’s part of me that’s like, maybe I should complain more and remind them that I’m still sick. And I’m like, well that’s just stupid. So even your own family, they don’t see all the stuff”* (P02).

In addition to receiving negative appraisals in their personal lives, Long COVID patients also noted having these experiences in the workplace, which potentially impacted their self-evaluation as professionals and even their job security. Although many employers initially agreed to provide work accommodations for Long COVID patients, some participants reported that these agreements were later retracted or blatantly ignored in practice. In these instances, employees with Long COVID were verbally or non-verbally appraised as underperforming and unreasonable for requesting continued support. One participant reflected, “*Initially, my boss was very supportive and when I came back, he was very supportive. When it came to performance review time, I do feel like he kind of screwed me over on that by listing my work as below, like as not meeting standards in any way, stating that it’s because of COVID*” (P14).

### 4.5. Belonging Support

Two themes emerged within the belonging support dimension: (1) positive belonging support through validating feedback and (2) barriers to seeking belonging support. Participants reported receiving belonging support from both their support groups and professional networks. One participant described how the validating feedback made them feel like part of a larger community of Long COVID patients: *“But there [Long COVID groups] are things that makes me feel like I’m not crazy. Because there are so many of us out here”* (P11).

At the same time, some Long COVID patients noted barriers that hindered them from seeking such support, such as their lack of desire to participate in social situations. One participant shared their experience: “*I guess that because I’m not in society, I guess it’s hard to have a stigma you know, because I’m not out there, interacting with society. Really, I’m home ninety percent of the time*” (P07). Furthermore, the physical and mental fatigue caused by Long COVID may have made it difficult for some participants to even contemplate socializing. One participant noted, “*Even when they opened back up, I have no desire to go to a restaurant, to go to any social. I have not been to a wedding, a funeral, or a birthday party for my family since having COVID. And I lost one really good friend to COVID, and I didn’t even go to her funeral. I just don’t want to be around a lot of people*” (P21).

## 5. Discussion

Our study illuminates the types of social support Long COVID patients perceived they had received in their work and personal lives, in alignment with all five dimensions of the Social Support Framework [32,33]. While emotional, tangible, and informational support were generally described in positive terms, Long COVID patients also reported receiving negative appraisal support, which potentially impacted their self-esteem both personally and professionally. Experiences characterized within the belonging support dimension were mixed, with some participants expressing a sense of connectedness through receiving feedback that validated their identity as working professionals or as individuals living with a chronic illness, which is not readily apparent. Consistent with previous literature, individuals with high levels of social support are more likely to receive emotional validation, understanding of their situation, and tangible assistance in coping with their experiences. However, other participants emphasized feelings of alienation they attributed to having Long COVID. More specifically, given the nature of this invisible chronic illness, members of some patients’ social networks lacked understanding about their unique experiences. This made it difficult for Long COVID patients to seek out certain forms of social support, such as belonging support. While high levels of social support have been found to reduce feelings of alienation, alienation among this group was found to be a barrier to Long COVID patients receiving forms of social support. Notably, this experience of “otherness” has been described in the broader literature on chronic illness, as patients leading restricted lives often become consumed by the physical and psychological burdens of their condition [36,37,38,39].

Our study also provided insight into the social support needs of Long COVID patients and shed light on the types of social support that they reportedly found helpful. A tangible social support need consistently described by employed participants was flexibility in work arrangements. Emotional support has also been shown to play a critical role in the well-being and distress of Long COVID patients [40] and our study characterized different types of emotional support, such as check-ins, verbal encouragement, and expressions of understanding, that were reportedly helpful in supporting their emotional well-being as well as their coping with distress in times of hardship. Our study further delineated the different sources of emotional support, highlighting that Long COVID patients can receive helpful emotional support from the workplace in addition to from their family and friends. When employers foster a trusting and safe environment and show understanding about fatigue and the cognitive impairments commonly experienced by individuals with Long COVID, their employees faced with these challenges are better able to communicate their needs, which is an important factor for those with a chronic illness that may have invisible symptoms [41].

It is important to note that given the prolonged nature of Long COVID, this may require understanding in the provision of these forms of social support over an extended period. Patients in our study reported instances of social support waning or declining over time. This pattern aligns with previous literature, which suggests that chronic stressors associated with long-term illnesses can lead to the withdrawal of social support [42]. As support deteriorates and previously ailing individuals shift attitudes, patients with chronic illness may experience increased psychological distress. The existing literature also brings attention to the psychological impact of living with chronic illnesses, which has significant impacts on elements of self-esteem and self-image. Notably, negative social support is found to be a significant predictor of patients’ self-esteem over time [43]. These increases in distress and reductions in self-esteem may, in turn, hinder patients’ abilities to integrate effectively into their social and work environments.

A growing body of the literature has explored how personnel in the workforce act as either support systems or obstacles for individuals who continue working despite experiencing Long COVID symptoms that impair their functioning [15,44,45,46,47]. Our study adds to the literature by providing evidence of unhelpful appraisal support and identifying unmet needs in this dimension within the workplace. The appraisals perceived by our employed participants were overwhelmingly negative, including both verbal and nonverbal negative evaluations. Notably, participation in valued activities through employment is an important need for individuals with chronic illness and serves as a key reference point for building a sense of value, worth and capabilities; this is at odds with instances where employees reported being appraised at the workplace as unreasonable, underperforming, or incompetent through either negative performance reviews or rejection of work accommodation requests [48]. Our similar findings underscore this issue for Long COVID patients who may receive negative appraisals that may diminish their self-esteem as professionals.

Another previous study of chronic illness found that fatigue and symptom burdens made these patients feel that they did not physically have the capacity to seek help [44]. Expanding on this, findings from our study suggest that barriers to seeking support, especially in terms of belonging support, exist and may extend beyond physical limitations. Feelings of being alienated from society stemmed from the isolation associated with Long COVID and were reportedly compounded by reduced physical and mental energy, creating considerable obstacles for the patients to engage in social environments that might otherwise foster a sense of connection. This finding highlights a paradox: when connection may be most needed to counteract isolation and bolster diminished self-worth caused by negative appraisals, the very nature of Long COVID symptoms might prevent individuals from seeking or sustaining the relationships that could provide that support. Moreover, research has shown that the perception of being a burden can affect individuals with chronic illnesses by influencing their willingness to seek or accept social support [49]. This self-perception may serve as an additional barrier as patients ignore or conceal their needs in an effort to avoid imposing on those in their close social circles, which potentially contributes to their feelings of isolation and unmet support.

Our findings highlight not only the types of helpful social support Long COVID patients report receiving but also those unhelpful and unmet social support needs they experience, especially in the workplace. Furthermore, they point to clear opportunities for caregivers and employers to intervene and provide the types of social support that are currently lacking for individuals with chronic illnesses, who are challenged in navigating their personal and professional lives. Those who are chronically ill as a group experience work disparities in terms of the likelihood of unemployment and access to full-time work [50]. Individuals with Long COVID represent an emerging population facing similar work-related disparities. Consistent with the previous literature [51], our findings show that these disparities often manifest in inconsistent or absent workplace accommodations and a lack of recognition of patients’ chronic symptoms. Creating a trusting environment is essential for employees’ well-being [52], and our findings emphasize that such an environment can also help Long COVID patients feel safe expressing their need for support in the workplace. Efforts should aim to foster inclusion and support the return to work and retention of Long COVID patients. Employers can play a vital role by initiating open communication with affected employees to determine reasonable leave durations, return-to-work performance goals, and flexible accommodations. Strategies to facilitate such communications may include awareness training measures addressed to employers and managers to enhance understanding of the chronic and often disabling nature of Long COVID [44].

Our findings provide support for the use of the Social Support Framework in exploring the way social support influences the experiences of Long COVID patients. This framework, previously applied to a range of chronic illnesses such as type 2 diabetes [53], rheumatoid arthritis [54], and congenital heart disease [55], has been shown to be effective. While our use of the framework confirmed its relevance and extended its application to a new and underexplored population, the findings of this study also provide evidence to support the extension of this framework. For example, the emergence of negative appraisal support and barriers to belonging support highlight dimensions of social support that are not fully captured in the current model. These findings point to the value of considering the quality of support (e.g., when appraisal becomes judgmental rather than affirming) and the conditions under which support becomes inaccessible due to physical and mental fatigue associated with a chronic illness. In doing so, our study offers insights that may contribute to the theoretical expansion of the Social Support Framework to better account for the complexities of chronic illness experiences such as those observed among patients with Long COVID.

## 6. Limitations

Our study has several limitations. First, our data were drawn from patients at a single specialized clinic. This limits our ability to capture the experiences of the broader community, particularly those who may not have access to care or do not have the physical and/or mental energy to seek such care. Even within our study population, however, we found that some participants reported experiencing symptom burdens so overwhelming that their symptoms occasionally prevented them from seeking social support. Second, the majority of our sample experienced severe Long COVID symptoms for more than one year, which could have had a greater impact on their function in personal and professional lives. As a result, our findings may not apply to individuals with milder or shorter-term forms of Long COVID. Third, we did not systematically assess participants’ specific symptoms or pre-existing health conditions, which limited our ability to explore how symptom burden might influence both the types and amounts of social support received. Fourth, while the amount of our incentive was not exorbitant given the time required to participate in the study, there is a potential for self-selection bias. For example, the perspectives of patients who agreed to participate may differ from those who declined, potentially based on their illness burden, healthcare experiences, or satisfaction with care. Fifth, while broad generalizability is not the primary aim of our qualitative study, the findings may offer transferable insights into similar social contexts. Additionally, our demographic data collection was limited to age and gender, which prevented us from performing a more in-depth analysis of how other social determinants (e.g., race, socioeconomic status, and occupation) may shape differences in the types of social support received. We acknowledge that investigating differences across a broader range of sociodemographic characteristics is a valid direction for future research. Lastly, our study captured participants’ perspectives at a single point in time when the understanding of Long COVID was rapidly evolving. As public awareness and medical knowledge of the condition continue to grow, perceptions of social support may shift accordingly.

## 7. Conclusions

This study examined Long COVID patients’ perceptions of social support in both their work and personal lives. While participants reported receiving helpful and positive tangible, emotional, and informational support from a variety of sources, the appraisal support they described was often negative and, correspondingly, could undermine their self-evaluations as working professionals and as individuals managing a chronic and often invisible illness. These negative support experiences may potentially impact patients’ long-term self-esteem and their abilities to integrate effectively into their social and work environments. Despite some experiences of belonging support described, a number of patients reported that feelings of alienation and a lack of physical and mental energy to socialize prevented them from seeking belonging support. Greater awareness and broader public recognition of the chronic nature and symptom burden of Long COVID are needed to address the negative appraisal experiences and to facilitate patients’ abilities to seek and receive belonging support. Awareness training for employers focused on strategies to foster inclusion, return to work, and retention at work may help improve the quality of support provided to the growing number of individuals with Long COVID, who continue to face unique and unequal challenges in both personal and professional domains.

## Figures and Tables

**Table 1:** Long COVID patient participant characteristics (*N* = 21).

Demographic Characteristic	n (%)
Age group (years)	
18–44	7 (33)
45–54	7 (33)
55–64	5 (24)
65+	2 (10)
Sex	
Female	16 (76)
Male	5 (24)
Year of first COVID-19 infection	
2020	10 (48)
2021	6 (28)
2022	5 (24)

**Table 2:** Summary of the themes and subthemes within the Social Support Framework, supported by representative quotations.

Themes and Subthemes	Description	Representative Quotation
**Tangible Support**		
Positive tangible support from family	Regular and substantial assistance with daily activities and household responsibilities	…my mom was supportive. She would drive me to appointments initially when I wasn’t feeling well enough to drive. And I wasn’t able to focus or remember things so she would help write things down for me and help in that way. And my husband, he was supportive … he would make sure that if I couldn’t do something around the house, that he would help with those things since I wasn’t able to lift things. We have two pets. So, he would be often taking care of the pets when I was in bed sick. (P08)
Positive tangible support from friends	Periodic assistance with physically demanding tasks	I’ve had a few real close friends that’s been super, super supportive. The one guy … we raise sheep, but used to have 250 ewes. And now we’re down to, this past year we’re down to 60 ewes. And so, my mom she stepped in to do chores. But I had a real good friend. He stopped over at least once a week, check on the sheep, make sure thing’s going right. That was a big relief for me. (P13)
Positive tangible support from the workplace	Being open to flexible work location and hours of work	My job has always been pretty flexible and I majority work from home. So, I didn’t really have to be treated any differently than I already was, which was good. So, they were able to continue letting me work from home. They’re flexible with paid sick time, so I had a good experience with that part. (P18)
Positive tangible support from neighbors	Occasional assistance with patients’ family, property, or livelihood, including farm work	I had two neighbor ladies come to me and say, listen, this is what’s going to happen. We are taking over your mom and dad, so we’ll watch over them. We’ll make sure they got everything they need. They will be fine. If you want to call and check on, that’s fine. But first, you got to take care of you. Because you’re gonna drive yourself into the grave at this rate. You gotta stop. (P21)
**Emotional Support**		
Positive emotional support from friends	Being flexible and understanding about changes in social activity arrangements Checking in on well-being	They’ve been really flexible with me. Like, if I tell them I’m not feeling well, they will offer to reschedule things for me. Or if we were supposed to hang out and go do something, they’ll say, we can hang out at your house and have a movie night. … And just supportive of my situation with my family, reassuring me that I’m the one that is sick, and my family is not acting correctly. And just being emotionally supportive in that way. (P18) I’ve had support from my friends. One of my friends in Columbus, he always called my wife every day. He practically called every day, called my wife every day, you know, with what’s the prognosis. (P09)
Positive emotional support from family	Verbal encouragement Being understanding about patients’ difficulties	And my friends and family help me out, like, oh, how you feeling? Just checking up on me and just being cognizant of how I’m doing day to day. And like, also, oh, I’ve noticed your head is hurting, do you need to go to the store or something like that? I think I’ve had a lot of support. I’m really grateful. (P02) Well, my husband’s great, you know. My husband is fantastic. And my children, my children are adults and I’m, the ones that I’ve been close to, they’ve been very supportive and very helpful too, so that’s really good. I don’t, I would not have been able to function at all, I wouldn’t have much of a life at all, if it weren’t for my husband. He’s very understanding and a very hard worker and he doesn’t complain. And he helps me a whole lot because I really appreciate that. (P07)
Positive emotional support from the workplace	Creating a trusting, safe and understanding environment	He’s [my boss] real flexible and if something happens you know, it’s understandable and that’s life. Like things happen in life, at work. (P09)
**Informational Support**		
From credible sources	Medical information about COVID-19 and medications provided by online sources	I also went just through other resources that I’ve kind of kept in touch with, medical journals, different programs with OSU, and a couple of other hospitals (John Hopkins, and Henry Ford Health), and was able to find some articles that showed there was some relief on people taking Benadryl. (P03)
From support groups	Information about other patients’ experiences with Long COVID	But in this particular situation, I have found myself wanting to do something like that. And it’s been more about, I mean there’s validation there, right? But the other part is just having conversations with them. Say, hey you know, what are you doing to…? Oh, sleep disturbances, my sleep has been hideous. So, what are you doing to try to manage the sleep deprivation? Or what strategies are you doing to manage this? (P01)
**Appraisal Support**		
Negative appraisal support in personal life	Appraising patients as overreacting to their symptoms through explicit and implicit communication	She [my mom] believes it’s real, but she doesn’t believe it’s as bad as they say it is. … And [she] just telling me over and over again, it’s not that bad. (P18)
Negative appraisal support in the workplace	Evaluating patients as underperforming or unreasonable for requesting work accommodations	Unfortunately, after six months or so, I guess they got tired of it. And they started writing me up for the same things. They wrote me up for having brain fog. They were pushing to fire me, which made no sense. (P03)
**Belonging Support**		
Positive belonging support through validating feedback	Validating the existence of symptoms, affirming patients’ identities (e.g., identity as a patient)	And it makes me also feel grateful because so many Long COVID patients, their doctors are blowing them off and saying, because most of the time our tests come up normal. (P11) I just said [on LinkedIn], there was an issue with discrimination and former employer, and I’m being laid off and I’m looking to move forward to some place that is a better environment and a better culture. And I got a huge amount of response, not only from people that I’ve worked with in the past, but people I only know through LinkedIn that were networked. People throwing ideas out and mentioning my name. (P03)
Barriers to seeking belonging support	Feelings of alienation Lack of physical or mental energy to contemplate socializing	It’s the people that I know and the people that I care about that don’t understand. That’s the part that hurts the most. I don’t know if there’s anything that could help that, you know… (P07) It’s like a club I never wanted to belong to. But I don’t know what that would take to get people to understand that this can go on and on and on and on and on for months and months. And people just like, ehhhh, you could do it if you wanted to. There’s no reason why you can’t go out. There’s no reason why you can’t go to a crowded restaurant. Well, no, maybe there’s not, but I’m okay with not going. So why set my anxiety off by going? Maybe next month, I’ll want to, or the month after that. (P21)

## Data Availability

Data will not be made publicly available due to concerns for participant privacy.

## Supplementary Materials

The following supporting information can be downloaded at: https://www.mdpi.com/article/10.3390/healthcare13131568/s1.
