# Supplementary material for: Long COVID Patients’ Perceptions of Social Support in Their Work and Personal Lives: A Qualitative Study
Source: Healthcare (Basel). 2025 Jun 30;13(13):1568. doi: 10.3390/healthcare13131568 (PMC12249271; doi:10.3390/healthcare13131568)
Supplement: Supplementary file 1 [file healthcare-13-01568-s001.zip › healthcare-3626010-supplementary.pdf]

Code	Definition	Examples	What it is not
1-Background	Background information about respondent	County, age, urban/rural	
2-COVID-19 Experience	Acute episode of COVID-19	Description of when/how they got COVID, their experience when having acute COVID (e.g., time in hospital), their experience seeking care during acute COVID (e.g., visiting ER); may include other people's experiences with COVID-19 (for example, a family member's experience)	Not Long COVID discussion
3-Journey-Long COVID	Description of experience with Long COVID	Can include acute COVID and move into Long COVID	
4-Symptoms-Long COVID	Long COVID symptoms, experience of these symptoms over time; what most impacts life; change of symptoms over time	Fatigue, brain fog, cardiac symptoms, general feelings of being sick or feeling bad	Not experience with symptoms during acute COVID

5-Treatments-Long COVID	Treatments, experience with treatments over time, experiences with clinical providers in support groups	Medications, procedures, therapy (mental health/physical/occupational), experiences with diagnostic testing related to Long COVID (e.g., MRI, cognitive testing, cardiac testing)	Not treatments for acute COVID
6-Coping strategies	Strategies or methods to cope with a Long COVID symptom	Writing notes to help remember tasks to cope with brain fog, getting handicap parking placard to minimize fatigue from walking long distances, being active (e.g., bike riding)	Not treatments for Long COVID (medications, therapy)
7-Expectations-Long COVID	About when they will feel better from Long COVID, how this expectation has changed over time	e.g. "At first I thought I'd feel better in 2 weeks, then in a month. Now I'm not sure I'll ever feel the same as I did before COVID."	
8-Vaccines	Plans, perspectives, experience, politics related to COVID vaccine. Receipt of vaccine, booster, timing with Long COVID, impact of vaccine on Long COVID		

	symptoms, why not vaccine, politics about vaccine and process, trust		
9-Support-family and friends	Both support and where support was missing from family and friends	How family pitched in during experience with Long COVID. e.g. "Losing touch with friends that didn't understand that I wasn't feeling better."	Not support/lack of support from providers
10-Support-providers	Perceptions about support and lack of support from providers, care team, Long COVID clinic	Providers willing to try new treatments, providers listening to and believing symptoms. Perceptions of lack of support from providers, like not feeling good about being turned away from ER, primary care providers not taking Long COVID symptoms seriously.	Not support/lack of support from family/friends
11-Impact-employment/school	Support or lack of support from employer, co-workers, school, impact of Long COVID on employment	Understanding and mechanisms for help from employers/school. Lack of support, like losing a job or leaving school due to Long COVID experience/symptoms. Discrimination at work due to Long COVID symtoms (e.g., leading to termination)	
12-Impact-mental health/support groups	How has journey with Long COVID impacted your mental health, that of your family, attending support groups		

13-Impact-financial	Financial impact of Long COVID on self, family, change over time; role of insurance in paying/not paying for Long COVID related bills; impact on non-medical expenses (childcare, food, transportation, rent, other medications)	Costs related to Long COVID (or acute COVID). Impact on costs beyond medical expenses, like depleting savings, or needing to ask for financial help. Indirect costs related to Long COVID, like having to pay to have groceries delivered, have someone clean the house, mow the lawn, etc.	
14-Healthcare—skipped	Postponed or skipped medical appointments, due to finances	Long COVID treatments skipped due to cost, healthcare skipped for non-COVID related issues	
15-Isolation	Physical isolation, quarantine, seeing other people go back to normal when you have not, being alone, feeling separated	e.g. "Other people have moved on but I can't"	Not isolation from lack of support from family or friends

16-Loss	Sense of loss in identity, loss of sense of self, loss of who I used to be	Grief experienced due to loss of identify, self, who I used to be; e.g. "I liked being an active person, but now I don't have the energy"; difficulties with accepting loss of health (accepting sickness and new limitations in health); e.g. "ruined life"; loss of hope (double code with expectations); loss of identify related to body image (e.g., weight gain due to Long COVID, for example not being able to be active)	
17-Information sources	Information sources accessed by patients, trust in these sources, including between other Long COVID patients	Sources that are used or wanted; concerns about sources of misinformation	
18-Stigma	Responses to question about stigma (may overlap with support code); judgement from others; symptoms that feel especially linked to stigma	Feeling like they are judged because of their symptoms, having their symptoms not believed	
19-Concerns-Long COVID	Looking ahead, concerns about Long COVID	Reflections on long term disability and being able to care for self	

20-Informing Providers	What do you want doctors to know about being a patient with Long COVID		
21-ChitChat	Things not related to the interview topics	Thank you for participating, conversation with interviewee at end of interview	
22-Z-hold	Things that are important, but do not have a code		

Revised coding dictionary
